# Energy-Efficient Multi-Agent Deep Reinforcement Learning Task Offloading and Resource Allocation for UAV Edge Computing

**DOI:** 10.3390/s25113403

**Published:** 2025-05-28

**Authors:** Shu Xu, Qingjie Liu, Chengye Gong, Xupeng Wen

**Affiliations:** China Nanhu Academy of Electronic and Information Technology, Jiaxing 314001, China

**Keywords:** energy-efficient, unmanned aerial vehicles, task offloading, resource allocation, deep reinforcement learning, multi-agent systems

## Abstract

The integration of Unmanned Aerial Vehicles (UAVs) into Mobile Edge Computing (MEC) systems has emerged as a transformative solution for latency-sensitive applications, leveraging UAVs’ unique advantages in mobility, flexible deployment, and on-demand service provisioning. This paper proposes a novel multi-agent reinforcement learning framework, termed Multi-Agent Twin Delayed Deep Deterministic Policy Gradient for Task Offloading and Resource Allocation (MATD3-TORA), to optimize task offloading and resource allocation in UAV-assisted MEC networks. The framework enables collaborative decision making among multiple UAVs to efficiently serve sparsely distributed ground mobile devices (MDs) and establish an integrated mobility, communication, and computational offloading model, which formulates a joint optimization problem aimed at minimizing the weighted sum of task processing latency and UAV energy consumption. Extensive experiments demonstrate that the algorithm achieves improvements in system latency and energy efficiency compared to conventional approaches. The results highlight MATD3-TORA’s effectiveness in addressing UAV-MEC challenges, including mobility–energy tradeoffs, distributed decision making, and real-time resource allocation.

## 1. Introduction

The rapid evolution of UAV technology and the Internet of Things (IoT) has catalyzed the emergence of innovative applications such as virtual reality, high-definition video streaming, autonomous driving, and smart home systems [1]. These applications demand substantial computational resources and impose stringent latency requirements, while user expectations for data transmission speeds and service quality are growing exponentially. However, traditional mobile devices (MDs), constrained by limited computational power, storage capacity, and energy resources, often fail to meet these demands, hindering the widespread adoption of 5G technology. To address this, Mobile Cloud Computing (MCC) was introduced, leveraging centralized cloud service centers to provide shared resource pools. While MCC enhances data processing and storage capabilities, improving service reliability and efficiency, the physical distance between cloud centers and MDs often results in unstable communication connections and increased data transmission delays [2].

To overcome these limitations, Mobile Edge Computing (MEC) emerged as a transformative paradigm, deploying computational resources closer to MDs [3,4]. Since 2014, the European Telecommunications Standards Institute (ETSI) has been actively defining and releasing standard drafts for MEC, detailing its architecture and exploring its critical applications in the 5G era, such as optimizing mobile video QoS (Quality of Service), VR streaming, video surveillance, V2X (Vehicle-to-Everything) applications, and industrial control. These applications aim to reduce network latency and significantly enhance service quality. In 2016, ETSI rebranded MEC as Multi-Access Edge Computing, reflecting its expanded scope from traditional 3GPP cellular networks to include fixed networks, Wi-Fi, and other access networks. Compared to MCC, MEC deploys small-scale data centers within wireless access networks near users, offering lower latency and higher service quality. This proximity ensures faster mobile data (MD) transmission and mitigates network congestion, thereby improving overall resource utilization.

Unmanned aerial vehicles (UAVs) have emerged as a promising platform for MEC, offering unique advantages such as high mobility, flexible deployment, and the ability to provide on-demand computational services in remote or disaster-stricken areas. In single-UAV MEC systems, however, task offloading and resource allocation face challenges due to the limited number and compact distribution of MDs. In scenarios with sparse MD distributions, UAVs are constrained by battery life and computational capacity, making it difficult to provide effective services. To address this, multi-UAV MEC systems have been proposed, where multiple UAVs collaboratively provide computational offloading services to ground MDs. Studies on the tradeoff between latency and energy consumption in multi-UAV MEC systems have focused on weighted system-wide latency and energy consumption [5] or the latency and energy issues in communication and computation processes [6].

Recent advances in energy-aware scheduling for Mobile Edge Computing leverage optimization and machine learning to minimize system energy consumption across diverse scenarios. Key approaches include deep reinforcement learning for asynchronous task offloading [7], game-theoretic and iterative methods for multi-user resource allocation [8,9], alternating optimization in STAR-RIS-assisted systems [10], Lyapunov-based interference management [11], and Q-learning for dynamic multi-user environments [12]. These studies collectively demonstrate the effectiveness of model-driven and learning-based techniques in optimizing MEC energy efficiency, with reinforcement learning emerging as a particularly promising direction for adaptive resource management in complex edge computing networks. However, these algorithms are limited by resources such as communication, computing and energy, which makes it necessary to balance its own energy consumption while reducing the delay of task processing. How to reasonably formulate task offloading and resource allocation strategies to achieve the goal of balancing task processing delay and energy consumption of unmanned aerial vehicles is a challenging problem.

This paper investigates task offloading and resource allocation in multi-UAV MEC systems, aiming to minimize the weighted sum of task processing latency and UAV energy consumption. Given the non-convex nature of this problem and the inclusion of discrete variables, it is modeled as a Markov Decision Process (MDP). A MATD3-based task offloading and resource allocation algorithm is proposed, jointly optimizing MD selection, UAV mobility control, UAV CPU frequency adjustment, and task offloading allocation. This algorithm accelerates convergence, effectively reducing UAV energy consumption and task processing latency.

The contributions of this paper are summarized as follows:

(1) A comprehensive task offloading and resource allocation framework for multi-UAV mobile edge computing systems is proposed, addressing scenarios with sparse MD distributions. The framework integrates three key components: (i) a mobility model capturing UAV and MD dynamics, (ii) a communication model accounting for time-varying channel conditions, and (iii) a computational offloading model with resource constraints. This integrated approach enables the formulation of a joint optimization problem that minimizes the weighted sum of task processing latency and UAV energy consumption.
(2) By modeling the problem as a Markov Decision Process, a Multi-Agent Twin Delayed Deep Deterministic Policy Gradient for Task Offloading and Resource Allocation algorithm, namely, MATD3-TORA, is proposed to solve the optimization problem. The proposed solution incorporates three key innovations: (i) a twin-critic architecture to prevent overestimation bias, (ii) a delayed policy update mechanism for training stability, and (iii) a distributed execution framework that enables scalable coordination among multiple UAVs.
(3) Extensive experiments demonstrate that the proposed MATD3-TORA algorithm achieved superior performance compared to benchmark strategies on the weighted latency and energy consumption. The convergence performance of the proposed algorithm was analyzed under varying learning rates and exploration rates. Furthermore, the algorithm achieved the lowest weighted sum of task processing latency and UAV energy consumption across different computational task volumes and transmission bandwidths, validating its effectiveness.

The structure of this paper is organized as follows. Section 2 provides a comprehensive literature review, focusing on the application of deep reinforcement learning in UAV-enabled mobile edge computing systems. Section 3 presents the system models, including the mobility model, communication model, and computational model, which collectively form the foundation for the proposed framework. Section 4 elaborates on the novel Multi-Agent Twin Delayed Deep Deterministic Policy Gradient algorithm. Section 5 provides an in-depth analysis of the experimental results, including a discussion of the algorithm’s effectiveness in optimizing task offloading and resource allocation. Finally, Section 6 concludes the paper by summarizing the key findings, contributions, and potential future research directions.

## 2. Literature Review

In traditional Mobile Edge Computing systems, research on task offloading and resource allocation has primarily focused on various optimization objectives, including minimizing latency [13,14,15,16], reducing energy consumption [7,8,9,10,11,12], lowering costs [17,18,19,20], and maximizing utility [21]. Among these, minimizing latency and energy consumption has been a central focus.

Several studies have explored latency optimization in MEC systems, yet inherent limitations persist. Saleem et al. [13] proposed a Joint Partial Offloading and Resource Allocation (JPORA) scheme for the problem of minimizing total task processing latency in Device-to-Device (D2D)-enabled MEC systems, but its reliance on centralized optimization may hinder scalability in large-scale networks. Shu et al. [14] addressed the issue of minimizing expected execution latency for fine-grained tasks in low-power MEC systems and introduced a lightweight multi-user offloading strategy with a distributed consensus mechanism; however, their approach assumes ideal wireless conditions, neglecting dynamic interference fluctuations. Xiao et al. [15] explored the minimization of system latency in multi-user, multi-server MEC and decomposed the latency minimization problem into task offloading and energy allocation subproblems, solving them via matching theory and heuristics, yet their single-server-per-user constraint restricts flexibility in multi-server environments. Wan et al. [16] studied latency minimization in Non-Orthogonal Multiple Access (NOMA)-based MEC systems and developed a heuristic algorithm for NOMA-based MEC, combining binary search and swap matching, but the computational overhead increases exponentially with user density. Goudarzi et al. [22] proposed a distributed application placement framework leveraging an actor–critic reinforcement learning paradigm, specifically employing the IMPortance Weighted Actor Learner Architecture (IMPALA) to optimize resource allocation in edge computing environments. In a complementary study, Azizi et al. [23] proposed two novel semi-greedy heuristic algorithms—Priority-Aware Semi-Greedy (PSG) and its enhanced variant incorporating a multi-start procedure—to address the challenge of deadline-constrained IoT task scheduling in fog networks, demonstrating significant improvements in mapping efficiency.

Energy-efficient MEC strategies also exhibit critical tradeoffs. Chen et al. [7] addressed the problem of minimizing total system energy consumption in asynchronous task MEC systems, proposing a DDQNL-IST-based polling feedback energy-saving offloading strategy, yet their approach lacks adaptability to bursty workloads. Li et al. [8] tackled the minimization of total energy consumption for all users in multi-user, multi-server MEC systems, employing game theory and Lagrangian functions to solve the offloading strategy and resource allocation subproblems, but their Nash equilibrium-based solution may not guarantee global optimality. Wang et al. [9] investigated the minimization of total energy consumption for all mobile users (MUs) in multi-access MEC systems, introducing a two-layer iterative algorithm to jointly optimize task offloading rates, computational frequencies, and transmission precoding matrices for each MU. Zhang et al. [10] studied energy consumption minimization in Simultaneously Transmitting and Reflecting Reconfigurable Intelligent Surface (STAR-RIS)-assisted MEC systems, proposing an alternating algorithm to optimize STAR-RIS transmission and reflection coefficients, but their algorithm assumes static RIS configurations, limiting responsiveness to mobility. Sana et al. [11] addressed the problem of minimizing system energy consumption under inter-cell and intra-cell interference, formulating it as a long-term dynamic optimization problem and decomposing it into CPU scheduling and wireless resource allocation subproblems using Lyapunov stochastic optimization, yet their long-term dynamic formulation may not suit real-time applications. Zhou et al. [12] employed Q-learning and DDQN for joint offloading and resource allocation, but their model suffers from high training complexity in large state spaces.

In the context of latency and energy optimization in multi-UAV MEC systems, Liu et al. [24] studied task offloading in a system comprising high-altitude and low-altitude UAVs, where high-altitude UAVs optimized pricing to maximize revenue, while low-altitude UAVs minimized latency through task offloading. The authors proposed a multi-leader, multi-follower Stackelberg strategy to address this problem. Yang et al. [25] focused on minimizing system energy consumption in multi-UAV MEC systems, introducing a low-complexity iterative algorithm that jointly optimized user association, energy control, capacity resource allocation, and UAV positioning. This algorithm solved three subproblems and applied fuzzy c-means clustering to obtain feasible solutions. Zhang et al. [26] investigated the minimization of response latency in a multi-UAV MEC system consisting of a central top UAV and distributed bottom UAVs, employing stochastic geometric analysis of the three-dimensional UAV network and queuing theory to solve the problem. Tun et al. [27] addressed the problem of minimizing total energy consumption during task execution in multi-UAV MEC systems, proposing a block successive upper-bound minimization algorithm to jointly optimize offloading decisions, resource allocation mechanisms, and UAV trajectories. Almurairi et al. [28] tackled the minimization of UAV service latency in multi-UAV MEC systems, introducing a multi-layer edge cloud computing task offloading scheme based on integer linear programming. Zou et al. studied the minimization of total system latency in a multi-UAV MEC system integrated with Multiple-Input Multiple-Output (MIMO) technology, employing sequential convex optimization and block coordinate descent techniques to jointly optimize UAV trajectories and data offloading strategies.

Recent advancements in UAV-assisted edge computing have introduced innovative reinforcement learning approaches to optimize resource allocation and energy efficiency. For instance, Li et al. [29] developed a triple-learner-based reinforcement learning framework to simultaneously optimize UAV application placement and energy renewal. Addressing latency minimization, Liu et al. [30] employed DQN and DDPG algorithms to refine both UAV trajectories and virtual machine configurations. Wan et al. [31] introduced an online edge processing scheduling algorithm that dynamically adjusts task processing decisions based on real-time data rates to enhance computational efficiency. To address resource constraints, Wang et al. [32] integrated solar energy harvesting to power UAVs while providing computing services, thereby minimizing overall service costs. For high-dimensional continuous action spaces, Zhao et al. [33] leveraged a twin delayed deep deterministic policy gradient (TD3PG) algorithm to jointly optimize trajectory design, task allocation, and energy management. In the context of vehicular networks, Peng and Shen [34] utilized the MADDPG algorithm to manage multi-dimensional resources, maximizing the number of offloaded tasks. Similarly, Wang et al. [35] focused on energy efficiency, jointly optimizing UAV trajectories and computation offloading decisions while ensuring service fairness and collision avoidance. Seid et al. [36] proposed a DDPG-based scheme to minimize service costs and task execution delays by generating optimal computation offloading policies. Additionally, Liu et al. [37] introduced an attention-based MAPPO algorithm to optimize UAV computation offloading policies, minimizing weighted energy consumption while maintaining service fairness. To address resource allocation and energy efficiency in UAV-assisted networks, Ahmad et al. [38] developed a deep reinforcement learning framework to enhance spectral efficiency, network capacity, and resource distribution in beyond 5G networks, employing a deep energy-efficient resource allocation (EERA) method for dynamic and energy-conscious resource management. Nway et al. [39] proposed a block successive upper-bound minimization (BSUM) strategy to optimize resource allocation within a two-stage UAV-assisted edge computing system. Omoniwa et al. [40] introduced a communication-enabled multi-agent decentralized double-deep Q-network (CMAD-DDQN) approach to simultaneously improve energy efficiency and network coverage. These methodologies collectively address critical challenges in UAV-assisted networks, leveraging innovative optimization techniques to achieve enhanced performance.

From the above literature review, it can be found that current algorithmic approaches for MEC optimization exhibit methodological deficiencies, particularly in reinforcement learning (RL)-based methods applied to continuous action spaces. The pervasive issue of Q-value overestimation stems from inherent flaws in conventional Q-learning and DQN architectures, where the maximization bias in Bellman updates coupled with function approximation errors leads to systematic overestimation of the action values. This phenomenon critically undermines the stability and convergence properties of algorithms designed for fine-grained control tasks such as dynamic task offloading and resource allocation. In response to the above deficiencies, recent breakthroughs in RL theory have established dual-critic architectures (e.g., TD3/MATD3) and delayed update mechanisms as theoretically-grounded solutions to the Q-value overestimation problem. The twin Q-network paradigm fundamentally mitigates overestimation bias through independent value function training with minimum operator-based updates, while the policy delay mechanism prevents premature convergence by decoupling policy updates from value function optimization. These innovations are particularly salient for MEC systems requiring multi-agent coordination, as demonstrated by distributed task offloading in UAV swarms or collaborative resource allocation among edge servers. The MATD3 framework extends these advantages through its decentralized critic architecture and gradient delay mechanism, enabling stable optimization of continuous control variables in high-dimensional action spaces.

## 3. Problem Formulation

In this study, we consider a multi-UAV Mobile Edge Computing (MEC) system, where multiple UAVs operate in the air to provide computational offloading services for ground-based mobile devices. Existing research on the tradeoff between latency and energy consumption in multi-UAV MEC systems has been limited in scope. For instance, the authors of [5] focused solely on the weighted system-wide latency and energy consumption of computational offloading among UAV clusters, while [6] only addressed the latency and energy consumption associated with communication and computation within the system. Notably, none of these studies have considered the joint optimization of task processing latency and UAV energy consumption as a weighted sum.

To address this gap, this paper investigates the problem of task offloading and resource allocation in multi-UAV MEC systems, aiming to minimize the weighted sum of task processing latency and UAV energy consumption. Given the non-convex nature of this problem and the inclusion of discrete variables, we model it as a Markov Decision Process (MDP) and propose a novel task offloading and resource allocation algorithm based on MATD3-TORA. This algorithm jointly optimizes mobile device selection, UAV trajectory planning, UAV CPU frequency adjustment, and task offloading allocation.

### 3.1. Mobility Model

In the multi-UAV Mobile Edge Computing (MEC) system, we consider a scenario comprising *N* mobile devices (MDs) and *M* UAVs equipped with edge servers. Each MD has generated a set of computational tasks that exceed its local processing capabilities. To address this, MDs offload a portion of their tasks to UAV edge servers based on a predefined offloading ratio. Additionally, UAVs optimize their positions by selecting appropriate MDs and adjusting their locations to minimize the distance to MDs, thereby leveraging line-of-sight (LoS) channels to achieve enhanced channel gain and transmission rates.

The edge computing system, consisting of multiple MDs and UAVs, employs Time Division Multiple Access (TDMA) for communication between UAVs and MDs, while Frequency Division Multiple Access (FDMA) is used for communication among UAVs. The service period *T* is divided into *n* time slots, each with a duration of τ, during which only one MD occupies the channel to communicate with a UAV. At time slot *t*, the MD in the system is denoted as l∈{1,2,…,N}, with its coordinates represented as $z_{l}^{md}\left(t\right)={\left[x_{l}^{md}\left(t\right),y_{l}^{md}\left(t\right),0\right]}^{⊤}\in R^{3\times 1}$. Similarly, the UAV is denoted as u∈{1,2,…,M}, with its coordinates represented as $z_{u}\left(t\right)={\left[x_{u}\left(t\right),y_{u}\left(t\right),H\right]}^{⊤}\in R^{3\times 1}$, where *H* is the UAV’s altitude.

Given the stochastic mobility pattern where mobile devices (MDs) can randomly transition between spatial points within a given time interval, we employ the Gauss–Markov random mobility model to characterize this movement behavior. Specifically, the position of an MD at time slot *t* + 1 is updated according to

(1) $$x_{l}^{md}\left(t+1\right)=x_{l}^{md}\left(t\right)+v_{l}^{md}\left(t\right)cos\left(\theta_{l}^{md}\left(t\right)\right)\tau \;$$

(2) $$y_{l}^{md}\left(t+1\right)=y_{l}^{md}\left(t\right)+v_{l}^{md}\left(t\right)sin\left(\theta_{u}\left(t\right)\right)\tau$$

(3) $$z_{l}^{md}\left(t+1\right)={\left[x_{l}^{md}\left(t+1\right),y_{l}^{md}\left(t+1\right),H\right]}^{⊤}$$

In Equations (2) and (3), $v_{l}^{md}\left(t\right)$ and $\theta_{l}^{md}\left(t\right)$ represent the moving speed and direction of the mobile device UAV *l* in the time slot *t*, which can be expressed as

(4) $$v_{l}^{md}\left(t\right)=c_{1}v_{l}^{md}\left(t-1\right)+c_{2}\bar{v}+\sqrt{1-c_{1}^{2}\phi_{l}}$$

(5) $$\theta_{l}^{md}\left(t\right)=d_{1}\theta_{l}^{md}\left(t-1\right)+d_{2}\bar{\theta }+\sqrt{1-d_{1}^{2}\psi_{l}}$$

where $\bar{v}$ and $\bar{\theta }$ represent the moving speed and direction of the mobile device UAV *l* in the time slot *t*, and parameters $c_{1}$, $c_{2}$, $d_{1}$, and $d_{2}$ are used for evaluating the movement consistency of mobile devices UAV *l* within continuous time intervals. Meanwhile, $\phi_{l}$ and $\psi_{l}$ are two random variables that follow an independent Gaussian distribution, i.e., $\phi_{l}∼\left({\bar{\xi }}_{v_{l}},\zeta_{v_{l}}^{2}\right)$ and $\psi_{l}∼\left({\bar{\xi }}_{\theta_{l}},\zeta_{\theta_{l}}^{2}\right)$. These two distributions represent the randomness of the movement of the two devices.

For the multi-UAV MEC system, UAV *u* initiates its movement at the beginning of time slot *t* and updates its position by the start of time slot t+1 as follows:

(6) $$x_{u}\left(t+1\right)=x_{u}\left(t\right)+v_{u}\left(t\right)cos\left(\theta_{u}\left(t\right)\right)\tau_{fly}\;$$

(7) $$y_{u}\left(t+1\right)=y_{u}\left(t\right)+v_{u}\left(t\right)sin\left(\theta_{u}\left(t\right)\right)\tau_{fly}$$

(8) $$z_{u}\left(t+1\right)={\left[x_{u}\left(t+1\right),y_{u}\left(t+1\right),H\right]}^{⊤}\;$$

In Equations (6) and (7), $v_{u}\left(t\right)$ and $\theta_{u}\left(t\right)$ represent the velocity and direction of UAV *u* at time slot *t*, respectively, and $\tau_{fly}$ denotes the flight duration of the UAV.

This model ensures efficient task offloading and resource allocation by dynamically optimizing UAV trajectories and communication protocols, thereby enhancing the overall performance of the MEC system.

### 3.2. Communication Model

Assume that in a three-dimensional Cartesian coordinate system, the UAV maintains a constant altitude *H*, within which it can freely move on the corresponding horizontal plane. At time slot *t*, the initial coordinates of the UAV are denoted as $z\left(t\right)={\left[x\left(t\right),y\left(t\right),H\right]}^{⊤}\in R^{31}$, while its final position is represented as $z\left(t+1\right)={\left[x\left(t+1\right),y\left(t+1\right),H\right]}^{⊤}\in R^{31}$. The position of the mobile device (MD) *l* is given by $z_{l}^{md}\left(t+1\right)={\left[x_{l}^{md}\left(t+1\right),y_{l}^{md}\left(t+1\right),0\right]}^{⊤}\in R^{31}$. Thus, the Euclidean distance between UAV *u* and MD *l* at time slot *t* is expressed as

(9) $$d_{l,u}\left(t\right)=\sqrt{{\left(x\left(t\right)-x_{l}^{md}\left(t\right)\right)}^{2}+{\left(y\left(t\right)-y_{l}^{md}\left(t\right)\right)}^{2}+H^{2}}.$$

Let $\beta_{0}$ denote the channel gain at the reference distance d=1 m. For the line-of-sight (LoS) link, the channel gain between UAV *u* and MD *l* is given by

(10) $$h_{l,u}\left(t\right)=\beta_{0}/\left(d_{l,u}{\left(t\right)}^{2}\right).$$

In practical scenarios where obstacles may block the signal, the impact of such obstructions must be considered. The wireless transmission rate between UAV *u* and MD *l* is then expressed as

(11) $$r_{l,u}=Blog_{2}\left(1+P_{up}h_{l,u}\left(t\right)/\left(\sigma^{2}+P_{NLoS}b_{l,u}\left(t\right)\right)\right).$$

where *B* represents the signal bandwidth of the wireless communication, $P_{up}$ denotes the uplink transmission energy of the MD, $\sigma^{2}$ is the noise energy, and $b_{l,u}\left(t\right)$ is a binary indicator of whether an obstruction exists between UAV *u* and MD *l* at time slot *t*. Specifically, $b_{l,u}\left(t\right)=0$ indicates no obstruction, while $b_{l,u}\left(t\right)=1$ indicates the presence of an obstruction. $P_{NLoS}$ represents the energy loss due to non-line-of-sight (NLoS) conditions caused by obstacles such as trees or hills in the environment. This model captures the dynamic nature of UAV–MD communication, incorporating both LoS and NLoS conditions to ensure accurate representation of real-world scenarios.

### 3.3. Computational Model

In the multi-UAV Mobile Edge Computing (MEC) system, the offloading strategy adopts a partial offloading approach consistent with the single-UAV MEC system. Assume that mobile device (MD) *l* is served by UAV *u* during time slot *t*. Let $D_{l}\left(t\right)$ denote the total computational task volume of MD *l* at time slot *t*, and let $\alpha_{l,u}\left(t\right)$ represent the offloading ratio of tasks from MD *l* to UAV *u*. Consequently, $1-\alpha_{l,u}\left(t\right)$, u(t), represents the proportion of tasks processed locally. Thus, the local processing delay of MD *l* at time slot *t* can be expressed as

(12) $$t_{l,u}^{local}\left(t\right)=\left(1-\alpha_{l,u}\left(t\right)\right)D_{l}\left(t\right)S/f_{md},$$

where *S* denotes the number of CPU cycles required to process one unit of data, and $f_{md}$ represents the computational capability of the MD’s processor.

The processing delay for tasks offloaded from MD *l* to UAV *u* consists of two components—transmission delay and computational delay—formulated as follows:

(13) $$t_{l,u}^{tr}\left(t\right)=\alpha_{l,u}\left(t\right)D_{l}\left(t\right)/r_{l,u}\left(t\right),$$

(14) $$t_{l,u}^{comp}\left(t\right)=\alpha_{l,u}\left(t\right)D_{l}\left(t\right)S/f_{u}\left(t\right),$$

Since the computational results generated by MEC are typically small, the transmission delay of returning results via the downlink is negligible. In Equations (6)–(11), UAV *u* can dynamically adjust its operating frequency using Dynamic Voltage and Frequency Scaling (DVFS) technology. The CPU frequency of UAV *u* at time slot *t*, denoted as $f_{u}^{uav}\left(t\right)$, is given by

(15) $$f_{u}\left(t\right)=k_{u}\left(t\right)f_{max}^{uav},$$

where $f_{max}^{uav}$ is the maximum CPU frequency of the UAV, and k(t) is the CPU frequency adjustment factor at time slot *t*.

For the task volume $D_{l}\left(t\right)$ generated by MD *l*, the total processing delay is determined by the maximum of the local processing delay and the UAV processing delay. Therefore, the processing delay of UAV *u* for MD *l* is defined as

(16) $$t_{l,u}^{de}\left(t\right)=max\left\{t_{l,u}^{local}\left(t\right),t_{l,u}^{tr}\left(t\right)+t_{l,u}^{comp}\left(t\right)\right\},$$

For multiple UAVs at time slot *t*, the overall task processing delay is determined by the maximum delay among all UAVs. Thus, the task processing delay at time slot *t* is defined as

(17) $$t^{delay}\left(t\right)=max\left\{t_{l,u}^{delay}\left(t\right)\right\},\forall u\in \left\{1,2,\ldots ,M\right\},$$

The energy consumption of UAVs consists of two components: flight energy consumption and energy consumed by the MEC server during task computation. Let $M_{uav}$ denote the mass of the UAV. At time slot *t*, UAV *u* flies at a constant speed $v\left(t\right)\in \left[0,v_{max}\right]$, with an adjustment angle $\theta_{uav}\in \left[0,2\pi \right]$, moving from its initial position to its final position over a duration $\tau_{fly}$. The energy consumed during this flight is given by

(18) $$e_{fly}\left(t\right)=\phi {\left\|v_{u}\left(t\right)\right\|}^{2},$$

where $\phi =0.5M_{uav}\tau_{fly}$ represents the energy consumption coeffient. Additionally, the energy consumed by the MEC server of UAV *u* during computation is

(19) $$p_{u}\left(t\right)=\kappa {\left[f_{u}\left(t\right)\right]}^{3},$$

where κ is a constant coefficient. Therefore, the energy consumed by UAV *u* for computing tasks offloaded from MD *l* at time slot *t* is

(20) $$e_{comp}\left(t\right)=p_{u}\left(t\right)t_{l,u}^{comp}\left(t\right)=\kappa {\left[f_{u}\left(t\right)\right]}^{2}\alpha_{l,u}\left(t\right)D_{l}\left(t\right)S.$$

Given the small data size of computational results, the energy consumption for transmitting results back to the MD is negligible [41]. Thus, the total energy consumption of UAV *u* at time slot *t* is

(21) $$e_{to}\left(t\right)=e_{fly}\left(t\right)+e_{comp}\left(t\right),$$

In summary, the total energy consumption of all UAVs is

(22) $$E_{to}\left(t\right)=\sum_{t}\sum_{u}e_{to},$$

This model provides a comprehensive framework for optimizing task offloading and resource allocation in multi-UAV MEC systems, balancing both processing delays and energy consumption.

### 3.4. Mathematical Formulation

Assume that at time slot *t*, the computational task generated by a mobile device is divided into two parts based on the offloading ratio: one part is processed locally on the device, and the other part is offloaded to the UAV server for processing. These two parts are executed in parallel. This implies that, during a specific time slot *t*, the task processing delay consists of two components: the delay for local processing and the delay for offloading the task to the UAV edge server and processing it on the server. In the task processing workflow, the energy consumption of UAVs is divided into two major components: mobility energy consumption and computational energy consumption. Mobility energy consumption is primarily influenced by the UAV’s movement behavior, while computational energy consumption is mainly determined by the task volume and the UAV’s CPU frequency. Assume that each UAV *u* serves only one mobile device at each time slot *t*. Let $\eta_{l,u}\left(t\right)$ indicate whether mobile device *l* is served by UAV *u* at time slot *t*. The selection of mobile devices, UAV mobility, task offloading, and the UAV’s CPU frequency adjustment collectively impact both task processing delay and UAV energy consumption. Therefore, this study aims to jointly optimize mobile device selection, multi-UAV mobility control, UAV CPU frequency adjustment, and task offloading allocation, with the objective of minimizing the weighted sum of task processing delay and UAV energy consumption across all time slots while satisfying the total computational task requirements and service period constraints. The optimization problem is formulated as follows:

(23) $$min_{\eta_{l,u}\left(t\right),z_{u}\left(t+1\right),\alpha_{l,u}\left(t\right),k_{(}u)}\sum_{t=1}^{T}\sum_{l=1}^{N}\eta_{l,u}\left(t\right)\left[w_{0}t^{de}\left(t\right)+w_{1}e_{to}\left(t\right)\right]\;$$

(24) $$C_{1}:x_{u}\left(t\right),x_{l}\left(t\right)\in \left[0,L\right],\forall t,\forall l,\forall u,$$

(25) $$C_{2}:y_{u}\left(t\right),y_{l}\left(t\right)\in \left[0,W\right],\forall t,\forall l,\forall u,$$

(26) $$\;C_{3}:\eta_{l,u}\left(t\right)\in 0,1,\forall t,\forall l,\forall u,$$

(27) $$\;C_{4}:\sum_{t=1}^{N}\eta_{l,u}\left(t\right)=M,\forall t,\forall u,$$

(28) $$\;C_{5}:w_{0}+w_{1}=1,w_{0}\in \left[0,1\right],w_{1}\in \left[0,1\right],$$

(29) $$C_{6}:{\left\|z_{u}\left(t\right)-z_{v}\left(t\right)\right\|}^{2}\neq 0,\forall u\neq v,$$

(30) $$C_{7}:b_{l,u}\in 0,1,\forall u,\forall l,\forall t,\;$$

(31) $$C_{8}:\sum_{t=1}^{T}e_{u}^{to}\left(t\right)\leq E_{max},\forall u,$$

(32) $$C_{9}:0\leq \alpha_{l,u}\left(t\right)\leq 1,\forall t,\forall l,\forall u,$$

(33) $$C_{10}:\sum_{t=1}^{T}\sum_{l=1}^{N}\sum_{u=1}^{M}\eta_{l,u}\left(t\right)D_{l}\left(t\right)=D_{max}$$

The constraints are defined as follows:

- Constraints $C_{1}$ and $C_{2}$ specify that the horizontal and vertical coordinates of UAVs and mobile devices must not exceed the predefined geographical boundaries, ensuring that all devices operate within the designated area.
- Constraint $C_{3}$ indicates whether mobile device *l* is served by UAV *u* at time slot *t*, where $\eta_{l,u}\left(t\right)=0$ denotes that mobile device *l* is not served by UAV *u*, and $\eta_{l,u}\left(t\right)=1$ denotes that mobile device *l* is served by UAV *u*.
- Constraint $C_{4}$ ensures that each UAV serves exactly one mobile device at any given time slot *t*.
- Constraint $C_{5}$ defines $w_{0}$ as the weight factor for task processing delay and $w_{1}$ as the weight factor for UAV energy consumption, with $w_{0}+w_{1}=1$. This constraint explicitly captures the tradeoff between processing delay and energy consumption.
- Constraint $C_{6}$ enforces that the positions of UAVs must not overlap, preventing potential collisions and crashes.
- Constraint $C_{7}$ describes the communication link obstruction between mobile device *l* and UAV *u*, where $b_{l,u}\left(t\right)=0$ indicates a line-of-sight (LoS) link, and $b_{l,u}\left(t\right)=1$ indicates a non-line-of-sight (NLoS) link.
- Constraint $C_{8}$ ensures that the total energy consumption of all UAVs does not exceed their maximum battery capacity $E_{max}$ over the entire service period.
- Constraint $C_{9}$ restricts the offloading ratio $\eta_{l,u}\left(t\right)$ to the range [0,1], where $\eta_{l,u}\left(t\right)=0$ represents full local execution, and $\eta_{l,u}\left(t\right)=1$ represents full offloading to the UAV edge server.
- Constraint $C_{10}$ guarantees that the total computational task volume $D_{max}$ is completed by the end of the service period.

This formulation provides a comprehensive framework for balancing task processing efficiency and energy consumption in multi-UAV MEC systems, ensuring optimal resource utilization and system performance.

## 4. The Proposed Algorithm

### 4.1. Markov Decision Process Modeling

The optimization problem is formulated as a Markov Decision Process (MDP), which comprises three core components: states, actions, and rewards. In the multi-UAV MEC system, at each time slot *t*, the UAVs interact with the environment, execute actions $A_{t}$ based on the current state $S_{t}$, receive rewards $R_{t}$, and transition to the next state $S_{t}^{\prime }$. Below, we detail the design of the state space, action space, and reward function for the multi-UAV MEC system.

#### 4.1.1. State Space

The state space in the multi-UAV MEC system includes UAV states, mobile device states, and channel states. Specifically, the UAV state is represented by its battery energy, position, and current CPU frequency. The ground environment state is characterized by the positions of all mobile devices, the remaining task volume, the randomly generated task volume of each mobile device, and the channel obstruction status between mobile devices and UAVs. Thus, the state of the UAV MEC system at time slot *t* is defined as

(34) $$s_{t}=\left(s_{1},\ldots ,s_{u},\ldots ,s_{M},s_{env}\right),$$

where $s_{1},\ldots ,s_{u},\ldots ,s_{M}$ represent the states of all UAVs, and $s_{env}$ denotes the task volume, mobile device positions, and channel obstruction status of the environment. Specifically, the UAV state and environment state at time slot *t* are expressed as

(35) $$s_{u}=\left(e_{u}\left(t\right),z_{u}\left(t\right),f_{u}\left(t\right)\right),$$

(36) $$s_{env}=\left(Z^{md}\left(t\right),D_{remain}\left(t\right),D^{md}\left(t\right),B^{md}\left(t\right)\right),$$

where $e_{u}\left(t\right)$ is the remaining energy of UAV *u*, $z_{u}\left(t\right)$ is the position of UAV *u*, $f_{u}^{uav}\left(t\right)$ is the CPU frequency of UAV *u*, $Z_{md}\left(t\right)=z_{1}^{md}\left(t\right),\ldots ,z_{N}^{md}\left(t\right)$ represents the positions of mobile devices 1 to *N*, $D_{remain}\left(t\right)$ is the remaining task volume of the system, $D_{md}\left(t\right)=D_{1}\left(t\right),\ldots ,D_{N}\left(t\right)$ denotes the task volumes generated by mobile devices 1 to *N*, and $B_{md}\left(t\right)=b_{1,u}\left(t\right),\ldots ,b_{l,u}\left(t\right)$ indicates the channel obstruction status between UAV *u* and mobile devices 1 to *l*. At the initial time slot (t=1), $e_{u}\left(t\right)=E_{max}$, and $D_{remain}\left(t\right)=D_{max}$. At the final time slot (t=T), $D_{remain}\left(t\right)=0$.

#### 4.1.2. Action Space

In the multi-UAV MEC system, continuous actions are related to mobile device selection, UAV mobility, UAV CPU frequency adjustment, and task allocation. Based on the observed environmental state, the action vector for all UAVs at time slot *t* is defined as

(37) $$A_{t}=\left(a_{1},\ldots ,a_{u},\ldots ,a_{M}\right),$$

(38) $$a_{u}=\left(l_{u}\left(t\right),\theta_{u}\left(t\right),v_{u}\left(t\right),\alpha_{l,u}\left(t\right),k_{u}\left(t\right)\right),$$

where $a_{1},\ldots ,a_{u},\ldots ,a_{M}$ represent the actions of UAVs 1 to *M*. For UAV *u*, the action $a_{u}$ includes the selected mobile device index $l_{u}\left(t\right)\in \left[0,N\right]$, the flight angle $\theta_{u}\left(t\right)\in \left[0,2\pi \right]$, the flight speed $v_{u}\left(t\right)\in \left[0,v_{max}\right]$, and the task offloading ratio $\alpha_{l,u}\left(t\right)\in \left[0,1\right]$. Similar to the single-UAV MEC system, the action space consists of continuous values, while the selected mobile device index $l_{u}\left(t\right)$ is discrete. Specifically, if $l_{u}\left(t\right)=0$, then $l_{u}=1$; if $l_{u}\left(t\right)\neq 0$, then $l_{u}=\left[l_{u}\left(t\right)\times N\right]$. Additionally, if mobile device $l_{u}$ is selected, the service indicator is defined as $\eta_{l,u}\left(t\right)=1$. The factor $k_{u}\left(t\right)$ adjusts the CPU frequency of UAV *u*.

#### 4.1.3. Reward Function

As analyzed above, minimizing the task processing delay and energy consumption of each UAV contributes to minimizing the overall objective function. Therefore, the reward for UAV *u* at time slot *t* is defined as $r_{u}$, which is the negative value of the weighted sum of task processing delay and energy consumption. A balancing factor ζ is introduced to normalize the scales of delay and energy. The reward function is formulated as

(39) $$R_{t}=\left(r_{1},\ldots ,r_{u},\ldots ,r_{M}\right),$$

(40) $$r_{u}=r\left(s_{u},a_{u}\right)=-\eta_{l,u}\left(t\right)\left[w_{0}t_{u}\left(t\right)+w_{1}e_{u}\left(t\right)\zeta \right].$$

This MDP framework provides a systematic approach to optimize task offloading and resource allocation in multi-UAV MEC systems, balancing both delay and energy efficiency.

#### 4.1.4. MATD3-TORA-Based Task Offloading and Resource Allocation Algorithm

**Figure 1:**
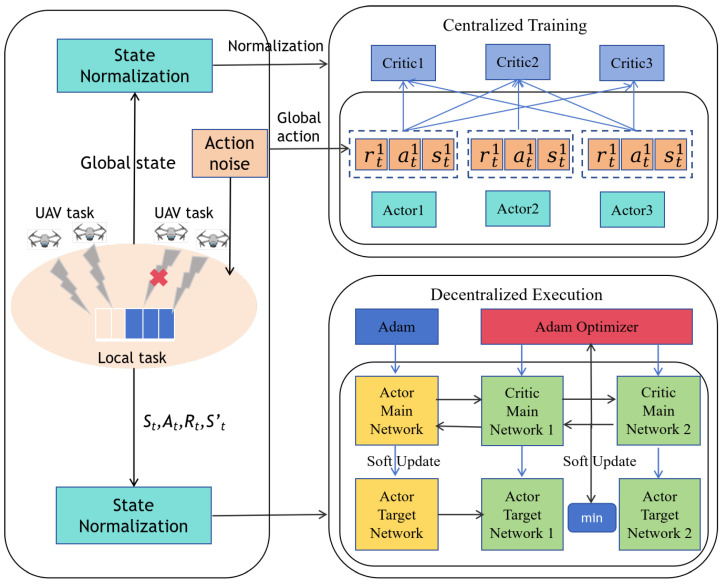
The MATD3-TORA algorithm framework.

After modeling the optimization problem as a Markov Decision Process (MDP), we integrate the MATD3 algorithm with the multi-UAV MEC system to propose the MATD3-TORA (Multi-Agent Twin Delayed Deep Deterministic Policy Gradient for Task Offloading and Resource Allocation) algorithm framework, as illustrated in Figure 1. Each agent is equipped with an independent actor network and dual-critic networks—consistent with the core design of the TD3 algorithm. The actor network generates actions based on the current state, while the dual-critic networks evaluate the expected returns of actions to guide the actor network’s updates.

Unlike the single-agent TD3 algorithm, the critic networks in MATD3-TORA consider the states and actions of all agents during evaluation. Specifically, the global state $S_{t}=\left(s_{1},\ldots ,s_{u},\ldots ,s_{M},s_{env}\right)$ and the global action $A_{t}=\left(a_{1},\ldots ,a_{u},\ldots ,a_{M}\right)$ are used to estimate the value of each actor network’s decisions. During training, each agent’s critic network predicts the expected reward for the agent given the current state and the actions of all agents. Although the input to the critic networks includes the states and actions of all agents, the output is an expected reward value specific to each agent, guiding the learning and optimization of its actor network. This approach allows each agent to optimize its policy based on the global environment state and the actions of other agents, ensuring coordinated and efficient strategies.

Based on this framework, we propose the MATD3-TORA algorithm, as outlined in Algorithm 1, which consists of the following eight steps:

(1) Initialization

Initialize the actor networks $\mu \left(s_{t}^{1}|\theta_{1}\right),\ldots ,\mu \left(s_{t}^{1}|\theta_{M}^{\mu }\right)$, the critic networks $Q_{1}\left(s_{t}\theta_{1}^{Q1}\right),\ldots ,s\theta_{M}^{Q1}$ and $Q_{2}\left(s_{t}\theta_{1}^{Q2}\right),\ldots ,s\theta_{M}^{Q2}$, and their target networks. Initialize the experience replay buffer *D* and the simulation parameters of the multi-UAV MEC system.

(2) Environment Reset

Reset the environment state $s_{t}$ of the multi-UAV MEC system and normalize the state $s_{t}$ using Algorithm 1 to obtain $s_{nor}$.

(3) Action Execution

Each UAV *u* selects its action $a_{u}$ based on the current policy $\mu (s_{nor}|\theta_{u})$ and Gaussian noise $n_{t}∼N\left(\mu ,\theta^{2}\right)$:

(41) $$a_{u}=\mu \left(s_{nor}|\theta_{u}\right)+n_{t}.$$

After executing action $a_{u}$, UAV *u* observes the next state $s^{\prime }$ and the immediate reward *r*. The transition tuple $\left(s,a_{u},r,s^{\prime }\right)$ is generated. Once all agents complete their actions and normalize their states, the global transition tuple $\left(S_{t},A_{t},R_{t},S_{nor}^{\prime }\right)$ is stored in the experience replay buffer.

(4) Sample Batch Extraction

Randomly sample a batch of $N_{b}$ transition tuples $\left(S_{b},A_{b},R_{b},S_{b}^{\prime }\right)$ from the experience replay buffer to update the actor and critic networks. For agent *u*, the corresponding sample is $\left(s_{u},a_{u},r_{u},s_{u}^{\prime }\right)$.

(5) Critic Network Update

For each agent *u*, MATD3-TORA employs a dual-critic structure to mitigate overestimation. The target Q-value $y_{u}$ is computed using the minimum of the outputs from the two critic networks:

(42) $$y_{u}=r_{u}+\gamma min_{i=1,2}Q\theta_{i}^{\prime }\left(s_{u}^{\prime },a_{u}^{\prime }\right),$$

where γ is the discount factor. The parameters of the critic networks are updated by minimizing the loss function:

(43) $$\theta_{Qi}\leftarrow \theta_{Qi}-\alpha_{critic}\nabla_{\theta_{Qi}}L\left(\theta_{Qi}\right),i=1,2,$$

where $\alpha_{critic}$ is the learning rate of the critic networks, and the loss function $L(\theta_{Qi})$ is defined as

(44) $$L\left(\theta_{Qi}\right)=1/N_{b}\sum_{b=1}^{N_{b}}{\left(y_{u}-Q_{\theta_{Qi}}\left(s_{u},a_{u}\right)\right)}^{2}.$$

(6) Actor Network Delayed Update

MATD3-TORA introduces a delayed update mechanism for the actor networks to reduce fluctuations caused by critic network estimation errors, thereby enhancing learning stability. The parameters of the actor network *u* are updated as

(45) $$\theta_{u}\leftarrow \theta_{u}-\alpha_{actor}\nabla_{\theta_{u}}J_{u},$$

where $\alpha_{actor}$ is the learning rate of the actor network, and the gradient $\nabla_{\theta_{u}}J_{u}$ can be computed as

(46) $$\nabla_{\theta_{u}}J_{u}=\nabla_{a}Q_{\theta_{Q_{1}}}\left(s_{u},a_{u}\right){|}_{a_{u}=\mu \left(s_{u}\right|\theta_{u}}*\nabla_{\theta_{u}}\mu \left(s_{u}|\theta_{u}\right),$$

(7) Target Network Update

MATD3-TORA employs soft updates to update the parameters of the target networks:

(47) $$\theta_{Q_{i}}^{\prime }\leftarrow \tau \theta_{Q_{i}}-\left(1-\tau \right)\theta_{Q_{i}}^{\prime },$$

(48) $$\theta_{\mu_{u}}^{\prime }\leftarrow \tau \theta_{\mu_{u}}-\left(1-\tau \right)\theta_{\mu_{u}}^{\prime },$$

where τ is a small constant close to 0, controlling the update step size.

(8) Iterative Update

Repeat Steps 2 to 7 until the total number of training iterations $I_{max}$ is reached. Finally, output the trained actor networks $\mu \left(s|\theta_{1}\right),\ldots ,mu\left(s|\theta_{M}\right)$.

This algorithm framework ensures efficient task offloading and resource allocation in multi-UAV MEC systems, balancing coordination, stability, and performance.
**Algorithm 1:** The MATD3 algorithm for task offloading and resource allocation.
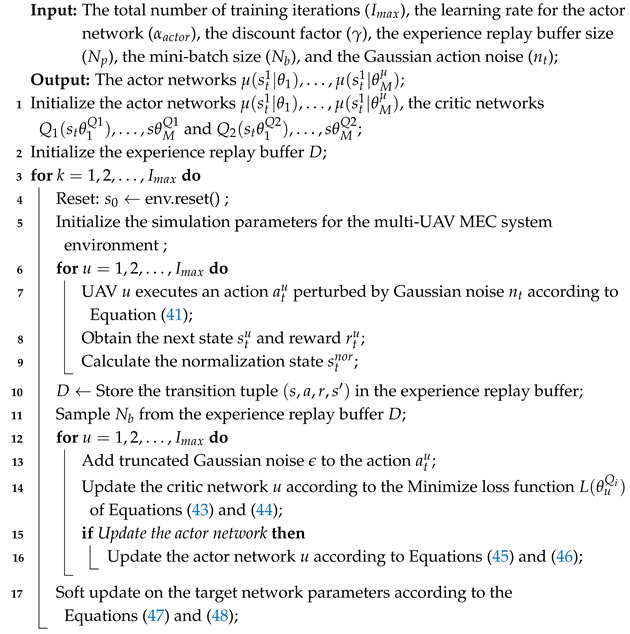


## 5. Experiments and Analysis

### 5.1. Experiment Settings

The experiments were conducted on a computational platform running Python 3.75 equipped with a 3.80 GHz CPU and 32 GB of RAM. The UAV simulation environment was configured within a three-dimensional space of 200 × 200 × 100 m. The total service period spanned 320 s, discretized into 40 time slots, each lasting 1 s. The UAVs were constrained to a maximum flight speed of 20 m/s. The transmission bandwidth was set to 1 MHz, with a channel gain of −50 dB at the reference distance of 1 m. The uplink transmission energy of the mobile devices was fixed at 0.1 W, while the non-line-of-sight (NLoS) penetration loss was set to 20 dB. The noise energy at the receiver was −100 dBm. Each UAV was equipped with a battery capacity of 500 kJ and a maximum CPU frequency of 1.2 GHz. The mobile devices operated at a CPU frequency of 0.6 GHz, with a computational requirement of 1000 CPU cycles per bit of data processed. The weight factors for delay and energy consumption were assigned values of 0.9 and 0.1, respectively. For the MATD3-TORA algorithm, the scaling factors for the state variables of individual UAVs were carefully determined. Specifically, the scaling factor for battery energy was set to the maximum battery capacity of the UAV. The scaling factors for UAV and mobile device positions were derived from the spatial limits of the ground environment, namely, its length and width. Additionally, the scaling factors for the total task volume and the task volumes of individual mobile devices were aligned with their respective upper bounds. The above parameters were rigorously selected based on well-established methodologies from the literature [42,43,44,45]. All environmental simulation parameters and their corresponding mathematical notations have been systematically defined to ensure reproducibility and consistency with prior research.

### 5.2. Algorithm Comparison

To effectively compare and evaluate the performance of the MATD3-TORA task offloading and resource allocation strategy in multi-UAV MEC systems, this study employed benchmark methods, including MAPPO [46] and QMIX [47]. All reinforcement learning algorithms underwent state normalization, as described below:

(1) Comparison with Edge-only

In this approach, the UAVs are stationed at fixed positions at the center of the region, functioning exclusively as Mobile Edge Computing servers for the mobile devices. At each time slot, all computational tasks generated by the mobile devices are offloaded to the UAVs for processing. This method relies entirely on the computational capabilities of the UAVs, with no tasks executed locally on the mobile devices. While this approach leverages the UAVs’ processing energy, it fails to optimize resource utilization and may lead to inefficiencies in task distribution.

(2) Comparison with Local-only

In contrast to the Edge-only method, this approach executes all computational tasks locally on the mobile devices without any assistance from the UAVs. The mobile devices rely solely on their own processing capabilities to complete all tasks. Although this method eliminates the need for task offloading, it is constrained by the limited computational resources of the mobile devices, resulting in higher delays and energy consumption for resource-intensive tasks.

(3) Comparison with Random

In this method, the UAVs are positioned at fixed locations at the center of the region and randomly select mobile devices and offloading ratios to process computational tasks. The movement of the UAVs and the selection of tasks are entirely random, lacking any optimization or strategic decision making. As a result, this method fails to achieve consistent performance and is highly inefficient in resource allocation.

(4) Comparison with MADDPG

The MADDPG algorithm, a well-established multi-agent reinforcement learning method, employs a single-target critic network to handle continuous action spaces. While it is suitable for multi-agent environments, it is prone to Q-value overestimation, which can hinder its convergence speed and optimization performance. Despite these limitations, MADDPG serves as a relevant benchmark for comparison due to its applicability to similar problem domains.

(5) Performance Analysis of MATD3-TORA

All reinforcement learning algorithms were trained for a total of 1000 iterations. As shown in the figure, the Random algorithm failed to converge due to its reliance on random selection or generation of solutions rather than deterministic rules or optimization processes. In contrast, both MADDPG and MATD3-TORA achieved convergence, with MATD3-TORA demonstrating superior performance. Specifically, this accelerated convergence of MATD3-TORA can be attributed to its innovative design, which addresses the issue of Q-value overestimation through the use of dual-critic networks, delayed policy updates, and target policy smoothing. These features enable MATD3-TORA to achieve more stable and efficient optimization compared to MADDPG.

Furthermore, the MATD3-TORA algorithm significantly outperformed the Edge-only and Local-only methods in terms of weighted delay and energy consumption. Specifically, MATD3-TORA achieved reductions in weighted delay and energy consumption, respectively. These results underscore the superior performance of MATD3-TORA in optimizing task offloading and resource allocation in multi-UAV MEC systems. By intelligently balancing the computational load between UAVs and mobile devices, MATD3-TORA minimizes delays and energy consumption while maximizing system efficiency.

In summary, the experimental results demonstrate that MATD3-TORA not only converges faster than MADDPG but also achieves significant performance improvements over traditional methods such as Edge-only and Local-only. Its ability to address Q-value overestimation and optimize resource allocation in a multi-agent environment highlights its potential as a robust and efficient solution for task offloading and resource allocation in multi-UAV MEC systems. These findings validate the superiority of MATD3-TORA and its applicability to real-world scenarios requiring dynamic and adaptive resource management.

### 5.3. Sensitivity Analysis

#### 5.3.1. Sensitivity Analysis for Learning Rates

**Figure 2:**
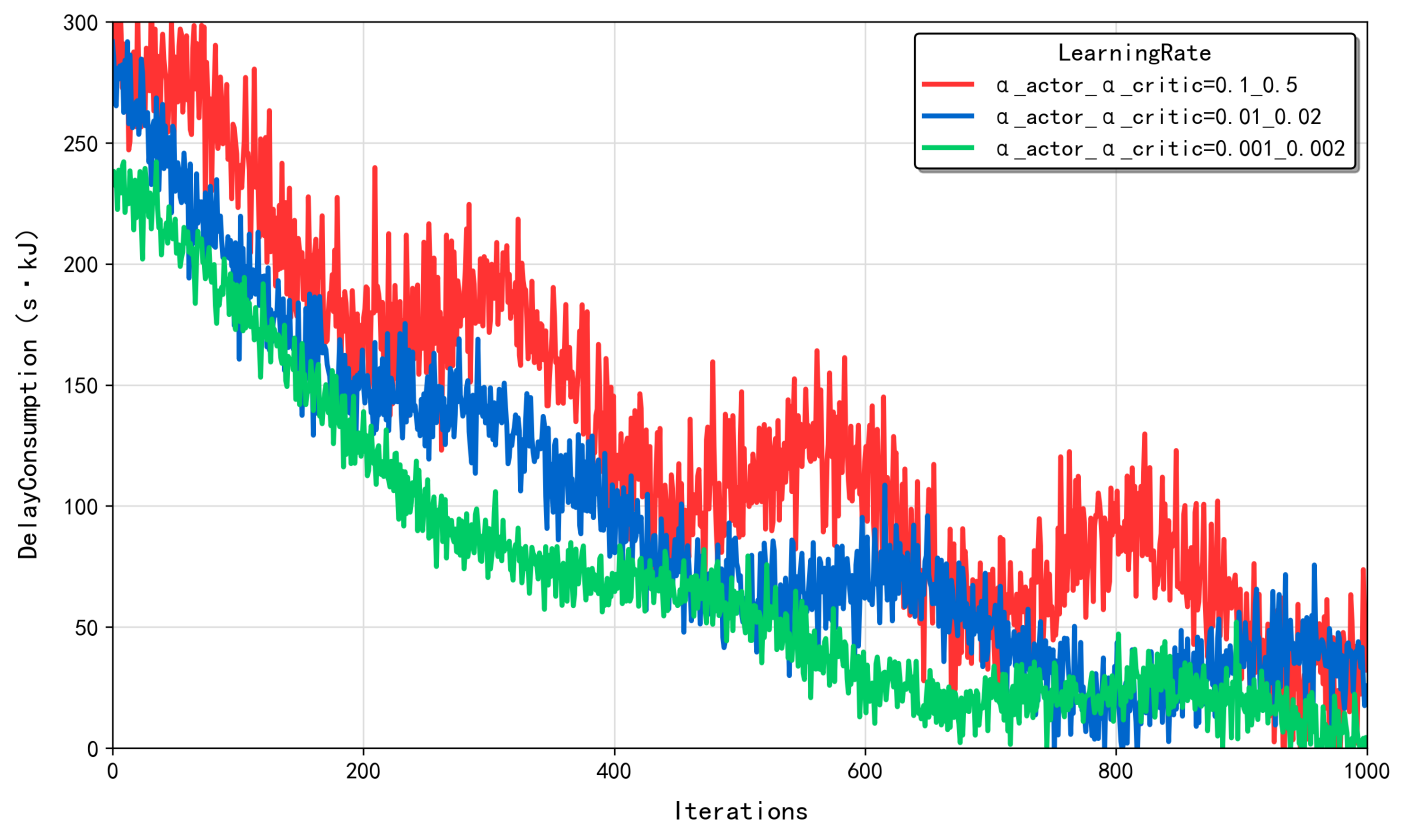
The convergence of the MATD3–TORA algorithm under varying learning rates α.

Figure 2 illustrates the convergence performance of the MATD3-TORA algorithm in a multi-agent environment under varying learning rate configurations. In actor–critic algorithms, the learning rates of the policy network (actor) and the value network (critic), denoted as $\alpha_{actor}$ and $\alpha_{critic}$, play a pivotal role in ensuring stability and convergence. Typically, these learning rates require meticulous tuning to adapt to diverse learning environments and tasks. For instance, $\alpha_{actor}$ and $\alpha_{critic}$ can be set to 0.1 and 0.5, smaller values such as 0.01 and 0.02, or even 0.001 and 0.002. As depicted in the figure, all three learning rate combinations achieved convergence. However, when $\alpha_{actor}$ and $\alpha_{critic}$ were set to 0.1 and 0.5 or 0.01 and 0.02, the algorithm converged to suboptimal solutions. This is primarily attributed to excessively high learning rates, which result in large update steps during iterations, causing the algorithm to overshoot the optimal solution. In contrast, when $\alpha_{actor}$ and $\alpha_{critic}$ were set to 0.001 and 0.002, the algorithm converged to superior results. This is because a moderate learning rate allows the algorithm to explore the solution space more thoroughly, enabling the discovery of better strategies. This analysis underscores the importance of carefully selecting learning rates to balance exploration and exploitation, thereby enhancing the algorithm’s ability to identify optimal policies in complex multi-agent environments.

#### 5.3.2. Sensitivity Analysis for Exploration Rates

**Figure 3:**
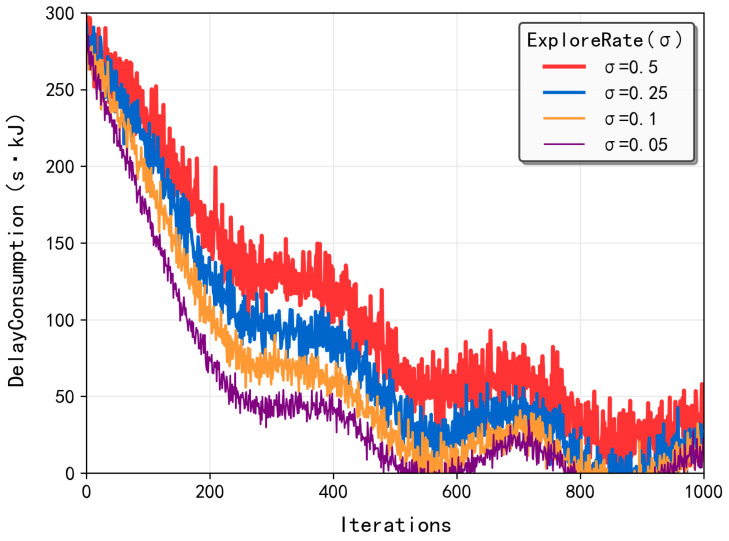
The convergence of the MATD3-TORA algorithm under varying exploration rates ($\sigma_{explore}$).

Figure 3 presents the convergence performance of the MATD3-TORA algorithm under varying exploration rates ($\sigma_{explore}$). The exploration rate was initialized at 1 and exponentially decayed with a fixed decay factor of 0.9997 per iteration until it reached the target value. When $\sigma_{explore}$ was set to 0.5, the algorithm relied heavily on random action selection for exploration, leading to convergence to a suboptimal solution. At $\sigma_{explore}$ = 0.25, the algorithm maintained a balance between exploring new actions and exploiting learned knowledge, reducing behavioral randomness and yielding solutions that approached the optimal solution. When $\sigma_{explore}$ was further reduced to 0.1, the algorithm, after sufficient exploration, predominantly exploited acquired knowledge to make decisions, resulting in convergence to the optimal solution. Notably, setting $\sigma_{explore}$ to 0.05 yielded results identical to those at 0.1, indicating that sufficient exploration had already been achieved, and further reduction in the exploration rate was unnecessary. This analysis highlights the critical role of the exploration rate in balancing exploration and exploitation, ensuring the algorithm’s ability to discover optimal strategies while minimizing unnecessary randomness in decision making.

#### 5.3.3. Sensitivity Analysis for Weights of Latency and Energy Consumption

**Figure 4:**
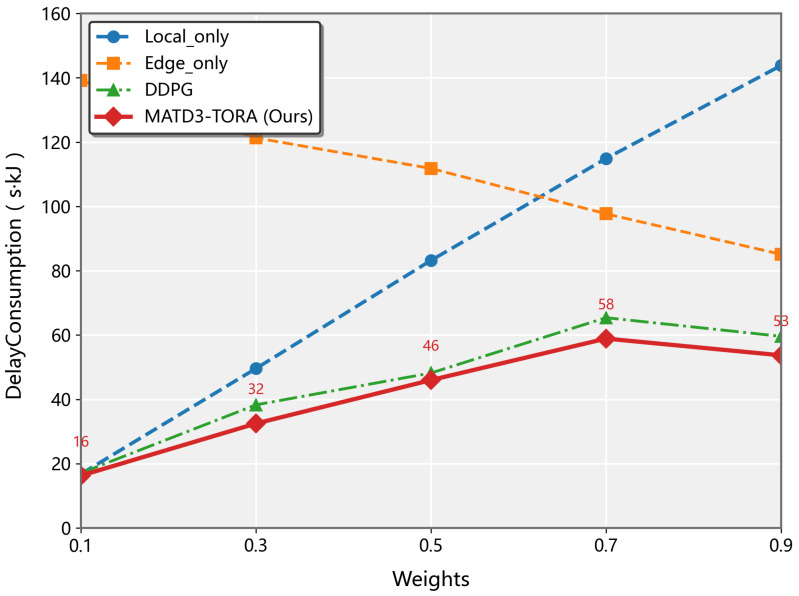
The convergence of the MATD3-TORA algorithm under varying weights (*w*).

As illustrated in Figure 4, the weighted cost (combining latency and energy consumption) exhibited distinct trends across different algorithms as the latency weight factor $w_{0}$ increased from 0.1 to 0.9. The Local-only strategy demonstrated a linear growth pattern (slope = 0.82), which is expected since it exclusively depends on local processing latency and remains unaffected by UAV energy consumption. Conversely, the Edge-only approach showed a decreasing trend (slope = −0.76) due to its complete reliance on UAV resources, making it more sensitive to energy consumption factors. The proposed MATD3 algorithm and baseline MADDPG approach both exhibited decreasing weighted costs, with MATD3 achieving superior performance. This improvement stems from MATD3’s optimized task offloading ratio that simultaneously considers both latency and energy factors. Specifically, while both algorithms are influenced by processing latency, MATD3 demonstrates better adaptability to the changing weight factors through its twin-delayed policy updates and more accurate value function estimation.

### 5.4. Performance Analysis

To comprehensively evaluate the performance differences between the MATD3-TORA task offloading algorithm and other benchmark methods, this section conducted a detailed analysis under varying total task volumes and transmission bandwidths. Notably, the Random algorithm was excluded from the analysis due to its failure to achieve convergence. Consequently, the comparison focused on four algorithms: MATD3-TORA, MADDPG, Local-Only, and Edge-Only. This approach ensured a rigorous and meaningful evaluation of the proposed algorithm’s effectiveness in diverse operational scenarios.

#### 5.4.1. Performance Variation of Algorithms Under Varying Total Task Volumes

**Figure 5:**
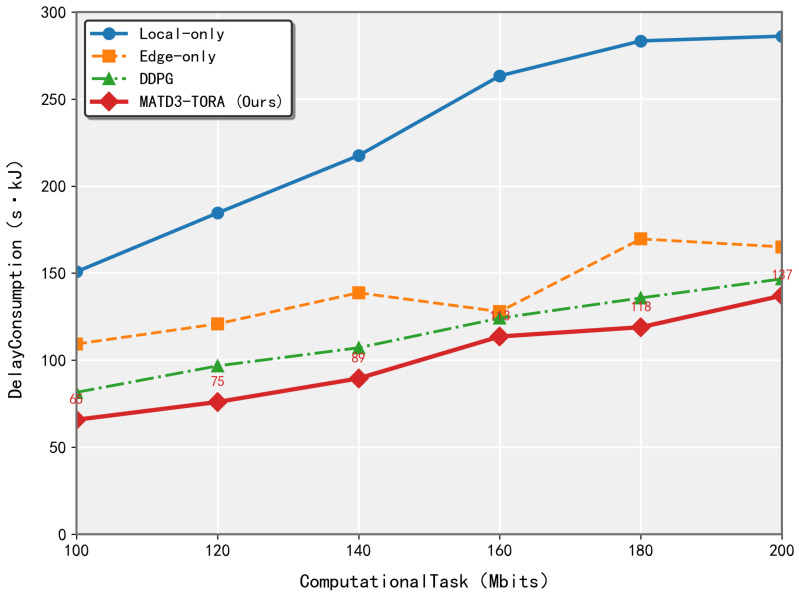
The performance variation of algorithms (MATD3-TORA, Edge-only, and MADDPG) under varying total task volumes.

As illustrated in Figure 5, the performance of the MATD3-TORA and MADDPG algorithms was evaluated as the task volume increased from 100 Mbits to 200 Mbits, with measurements taken at 20 Mbit intervals. The results demonstrate that both MATD3-TORA and MADDPG significantly outperformed the Local-Only and Edge-Only strategies in terms of weighted delay and energy consumption. Notably, the rate of increase in weighted delay and energy consumption for MATD3-TORA was lower than that of Local-Only and Edge-Only algorithms. This advantage stems from MATD3-TORA’s ability to determine optimal offloading ratios, thereby validating its effectiveness in task allocation. Although the convergence results of MADDPG and MATD3-TORA show minimal numerical differences, this is expected, since MATD3-TORA is an enhanced version of MADDPG, which is primarily designed to improve learning efficiency and stability rather than significantly altering the final outcomes. This analysis underscores the superior performance of MATD3-TORA in handling varying task volumes, highlighting its capability to achieve efficient and stable task offloading in dynamic environments.

**Figure 6:**
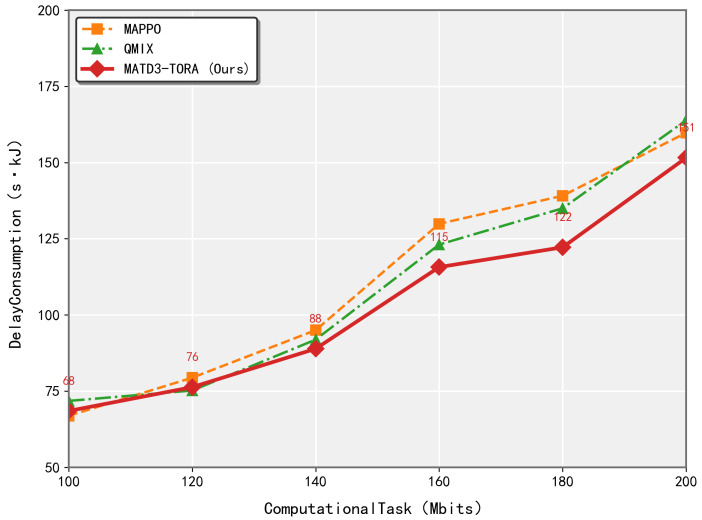
The performance variation of algorithms (MATD3-TORA, MAPPO, and QMIX) under varying total task volumes.

As seen in Figure 6, the proposed MATD3-TORA algorithm demonstrated superior adaptability and efficiency compared to MAPPO and QMIX when handling diverse task volumes. While existing methods struggle to maintain optimal offloading decisions under increasing computational loads, MATD3-TORA dynamically adjusts its resource allocation strategy through its hybrid decentralized–centralized architecture. This enables more effective balancing of latency and energy consumption across the UAV network. In contrast, MAPPO exhibits policy divergence under heavy task loads due to its unconstrained policy updates, while QMIX suffers from rigid value decomposition that fails to adapt to varying computation requirements. MATD3-TORA’s twin-delayed learning mechanism and task-aware optimization allow it to maintain stable performance across different workload intensities, whereas competing methods show significant degradation as task volumes scale.

#### 5.4.2. Performance Variation of Algorithms Under Varying Transmission Bandwidths

**Figure 7:**
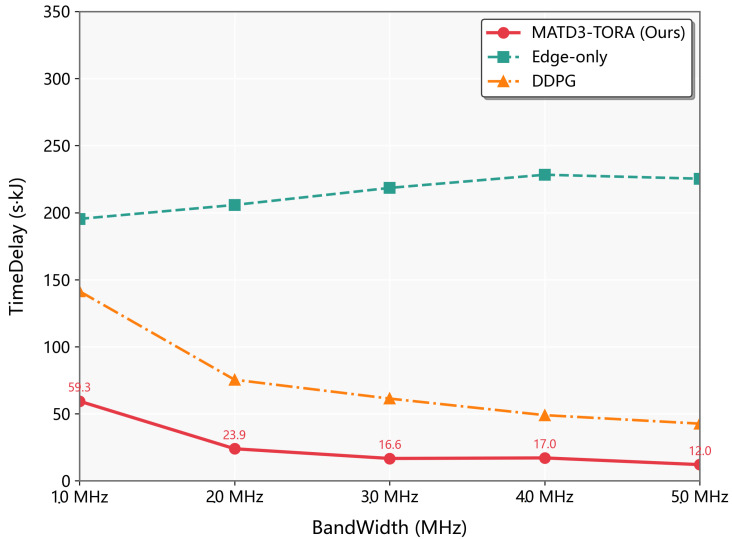
The performance variation of algorithms (MATD3-TORA, Edge-only, and MADDPG) under varying transmission bandwidths.

As depicted in Figure 7, the weighted delay and energy consumption of the Edge-only, MADDPG, and MATD3-TORA algorithms exhibited a decreasing trend as the transmission bandwidth increased from 1 MHz to 5 MHz. Notably, the Local-only algorithm is unaffected by changes in transmission bandwidth, since it does not involve task offloading via communication channels and was thus excluded from this analysis. The observed decline in the other three algorithms is attributed to the enhanced transmission rates resulting from increased bandwidth, which reduces the time required to transfer computational tasks to the UAVs, thereby lowering the weighted delay and energy consumption. This trend was particularly pronounced in the Edge-only algorithm, as it relies entirely on task offloading to the UAVs, making its performance more sensitive to variations in transmission rates. In contrast, the MATD3-TORA and MADDPG algorithms, through effective exploration, achieved optimal offloading ratios across different bandwidths. This adaptability is facilitated by the centralized training and decentralized execution mechanism of MADDPG, enabling these algorithms to operate efficiently in large-scale, high-dimensional, and dynamically changing environments. Consequently, MATD3-TORA and MADDPG were less susceptible to the significant performance fluctuations observed in the Edge-only algorithm when transmission rates varied. This analysis highlights the robustness of MATD3-TORA and MADDPG in optimizing task offloading under varying transmission bandwidths, demonstrating their superior adaptability and efficiency compared to the Edge-only approach.

**Figure 8:**
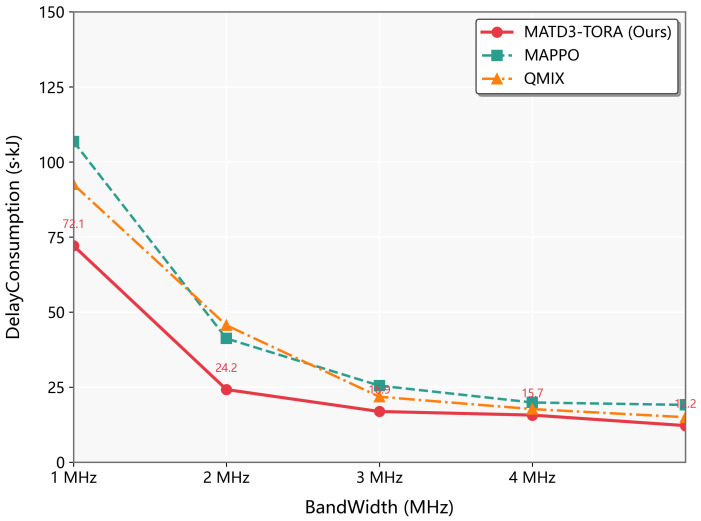
The performance variation of algorithms (MATD3-TORA, MAPPO, and QMIX) under varying transmission bandwidths.

As seen in Figure 8, it can be found that when subjected to different transmission bandwidths, MATD3-TORA outperformed both MAPPO and QMIX in maintaining efficient task offloading and network stability. The algorithm’s integrated channel-state awareness and distributed critic networks enable intelligent adaptation to bandwidth fluctuations, optimizing both communication and computation resources simultaneously. MAPPO’s performance varies substantially due to its sensitivity to partial observability in dynamic channel conditions, while QMIX’s centralized mixing network becomes a bottleneck under limited bandwidth. MATD3-TORA’s novel federated learning-inspired update scheme ensures robust decision making even in constrained bandwidth scenarios, preventing the performance collapse observed in baseline methods. This bandwidth-agnostic characteristic makes MATD3-TORA particularly suitable for UAV deployments where communication resources may vary unpredictably.

### 5.5. Limitations

While MATD3-TORA demonstrates promising performance in controlled UAV network scenarios, its scalability to large-scale deployments remains a critical limitation. The algorithm’s computational complexity grows exponentially with increasing network size due to its multi-agent decision-making architecture, potentially leading to impractical training times and real-time inference delays. Furthermore, the centralized critic component may become a bottleneck in distributed UAV networks with hundreds of nodes, as it requires global state information that becomes increasingly difficult to collect and process efficiently. The current formulation also assumes perfect communication links between agents, which may not hold in real-world large-scale operations where packet loss and latency variations are prevalent. These scalability challenges suggest the need for hierarchical architectures or distributed learning paradigms to enable MATD3-TORA’s application in massive UAV swarms while maintaining its coordination advantages.

## 6. Conclusions

This paper addresses the task offloading and resource allocation problem in multi-UAV mobile edge computing (MEC) systems, where UAVs provide communication and computational services to mobile devices. The study focuses on jointly optimizing mobile device selection, multi-UAV mobility control, UAV CPU frequency adjustment, and task offloading allocation to minimize the weighted sum of task processing delay and UAV energy consumption. To tackle this non-convex optimization problem, a novel task offloading and resource allocation algorithm based on MATD3-TORA (Multi-Agent Twin Delayed Deep Deterministic Policy Gradient for Task Offloading and Resource Allocation) has been proposed. The MATD3-TORA algorithm demonstrates superior performance by effectively reducing the weighted sum of task processing delay and UAV energy consumption, outperforming traditional benchmark methods.

Through extensive simulations, the convergence performance of MATD3-TORA was rigorously compared with benchmark methods, highlighting its faster convergence and greater stability. The algorithm’s robustness was further validated by analyzing its performance under varying learning rates, exploration rates, and training strategy parameters, which consistently show its adaptability to dynamic environments. Additionally, the study examines the algorithm’s performance under different task volumes, transmission bandwidths, and weight factors, demonstrating its ability to maintain optimal efficiency across diverse operational scenarios. The MATD3-TORA algorithm’s superiority lies in its ability to balance exploration and exploitation, leveraging dual-critic networks, delayed policy updates, and target policy smoothing to achieve more stable and efficient optimization. These features enable MATD3-TORA to outperform traditional methods such as Edge-only, Local-only, and MADDPG, particularly in complex and dynamic multi-UAV MEC systems. This research not only validates the effectiveness of the proposed algorithm but also provides valuable insights into its potential for real-world applications requiring adaptive and robust resource management.

## Data Availability

Data available on request due to restrictions.
